# Effectiveness and Productivity Improvement of Conventional Pultrusion Processes

**DOI:** 10.3390/polym14040841

**Published:** 2022-02-21

**Authors:** Evgeny Barkanov, Pavel Akishin, Endija Namsone-Sile

**Affiliations:** Institute of Materials and Structures, Riga Technical University, 1 Kalku Street, LV-1658 Riga, Latvia; pavels.akisins@rtu.lv (P.A.); endija.namsone@inbox.lv (E.N.-S.)

**Keywords:** pultrusion, finite element simulation, optimization, microwave heating, effectiveness, productivity

## Abstract

Pultrusion is a technological process in which fibers impregnated with resin move through the heated die and solidify into a composite profile with a constant cross section, as in the metallic die. The effectiveness and productivity of conventional pultrusion processes, preserving the quality of pultruded profiles, could be improved by process optimization or by the application of new, effective heating sources instead of electrical resistances with high heat losses. Due to the large dimension of the numerical problem and multiple iterations applied for the solution of government equations, an optimization methodology was developed, using the method of experimental design and the response surface technique. To develop microwave-assisted pultrusion processes, as well as pultrusion tooling design and process control, new effective electromagnetic-thermo-chemical finite element models and algorithms were developed by using general-purpose finite element software that results in considerable savings in development time and costs and makes available various modeling features of the finite element packages. The effectiveness and productivity of the optimized conventional pultrusion processes and the developed microwave-assisted pultrusion processes are estimated in comparison with the real pultrusion processes used in laboratory and industrial shops.

## 1. Introduction

Pultrusion is an automated technological process for the production of high-quality composite profiles with a constant cross section after the fibers’ impregnation with a resin and their movement through a heated metallic die [1]. The effectiveness and productivity of conventional pultrusion processes, reducing the cost of profiles, could be increased by process optimization or by the application of a new, effective heating source with less energy losses than those in present industrial processes.

Earlier, optimization problems were studied for a limited number of design parameters [2,3,4,5,6]. Later, the application of artificial neural networks, together with genetic algorithms [7,8], and planning of experiments and surrogate-based methodology [9,10] allowed increasing the number of investigated design parameters and solving more complicated optimization problems. A short review on the optimization of pultrusion processes can be found in [11]. However, in most optimization problems, steady-state thermo-chemical simulations are executed that do not allow estimation of the effectiveness of pultrusion processes with temperature control. Due to the limited number of optimization studies and some simplifications followed in numerical models, describing real pultrusion processes, the development of a more accurate and realistic optimization strategy; the examination of ambient room temperature, as an additional design parameter; and the use of a heater switch-on and switch-off strategy are required.

Among all possible heating methods, a high-frequency electromagnetic energy source [12] characterized by fast, instantaneous, non-contact, and volumetric heating can be examined as the best choice for pultrusion applications. It is necessary to note that now it is successfully used in industrial curing processes [13,14,15,16], and microwave-assisted pultrusion processes are analyzed only experimentally [17,18,19]. A limited number of studies [20,21] published recently on the simulation aspects of microwave-assisted pultrusion processes have slowed down their development. For this reason, new, reliable electromagnetic-thermo-chemical finite element models and algorithms were developed in this study by using the general-purpose finite element software ANSYS.

## 2. Optimization of Conventional Pultrusion Processes

**Figure 1:**
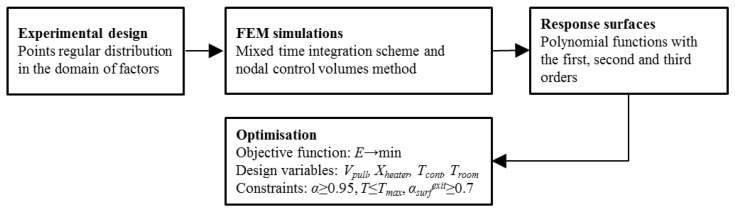
Optimization procedure.

Since it is necessary to operate with numerical problems of large dimension and considerable time is required for the solution of coupled thermo-chemical problems, an optimization methodology was developed using the method of experimental design [22] and the response surface technique [23] (Figure 1). In our case, distribution of the optimization variables in the design space was previously unknown. For this reason, the experimental plan with as regular as possible distribution of the points of experiments in the domain of factors was built in the first stage. Then, in each point of the plan of experiments, the coupled thermo-chemical problem was solved by the mixed time integration scheme and nodal control volume method [24]. This approach was validated successfully in an experimental trial by using cure sensors for the measurement of electrical resistivity and temperature on the profile surface [25]. More accurate and realistic process optimization was achieved with the algorithm developed in [26] and by allowing the temperature control executed by the heater switch-on and switch-off strategy. In the third stage, approximations were built using a conventional un-weighted least squares estimation with the elimination of some points [27], and polynomial functions with the first, second, and third orders were estimated in their applicability to describe the response surfaces. Finally, the constrained non-linear optimization problem was solved by the application of a new version of the random search method [28] and the generalized reduced gradient algorithm [29].

The objective function in the present optimization problems describes the effectiveness of pultrusion processes and minimizes the electrical energy (E) necessary for the production of a pultruded profile with the length of 1 m:

(1) $$E=\frac{n_{heater}\cdot W_{heater}\cdot k_{rt}}{m_{profile}\cdot V_{pull}}\to \min,$$

where $n_{heater}$ is the number of heaters, $m_{profile}$ is the number of simultaneously produced profiles, $W_{heater}$ is the power of the electrical heater, $V_{pull}$ is the process pull speed, and $k_{rt}={t_{work}/t}_{process}$ is the relative time of the heaters’ work during the manufacturing process [30], determined as the ratio between the time $t_{work}$, when the electrical heaters are switched on, and the full process time or simulation time, $t_{process}$. In general, the following variables are taken as the design parameters: pull speed $V_{pull}$, location of heaters $X_{heater}$, control temperature on heaters $T_{cont}$, and ambient room temperature $T_{room}$.

To achieve qualitative profile production, when the resin was fully cured, there was no overheating during the pultrusion process and it did not flow from the die, the following constraints were introduced: α≥0.95, $T\leq T_{\max}$, and $\alpha_{surf}^{exit}\geq 0.7$, where $\alpha =H(t)/H_{tr}$ is the degree of cure, H(t) is the amount of heat evolved during the curing up to time t, $H_{tr}$ is the total heat of reaction, T is the temperature, $T_{\max}$ is the maximal allowable temperature, and $\alpha_{surf}^{exit}$ is the degree of cure on the profile surface at die exit.

**Figure 2:**
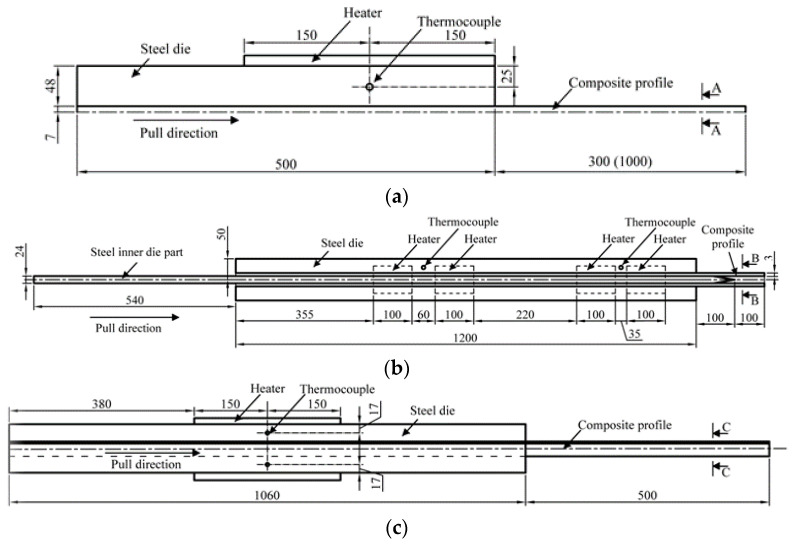

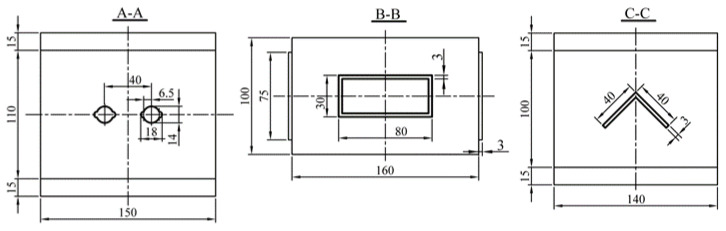
Schemes of pultrusion processes for (**a**) rod with ears profile, (**b**) thin-walled rectangular profile, and (**c**) corner profile.

The developed methodology was successfully applied for optimization of conventional pultrusion processes, producing profiles with different cross sections (Figure 2) and made of glass fibers and the following thermoset resins: polyester C-L ISO 112G, vinyl ester Crystic VE 676-03 [31], and epoxy Resoltech 1401+1407+AC140 [32]. The thermal properties of corresponding tool materials (Table A1) were taken from the handbooks and datasheets, but the properties of composite materials (Table A2) were measured experimentally with the lamped properties evaluated by the rule of mixtures [24]:

(2) $$\bar{\rho }=(1-V_{r})\rho_{f}+V_{r}\rho_{r}\;\bar{c}=\frac{(1-V_{r})\rho_{f}c_{f}+V_{r}\rho_{r}c_{r}}{\bar{\rho }}\;\bar{k}=\frac{k_{f}k_{r}\bar{\rho }}{(1-V_{r})\rho_{f}k_{r}+V_{r}\rho_{r}k_{f}},$$

where ρ, c, and k are the density, specific heat, and thermal conductivity, respectively; V is the volume fraction; and indexes f and r relate to the fibers and resin, respectively.

To describe the rate of resin reaction, the Kamal–Sourour curing kinetic model [33,34,35], which in the best way approximates the results of differential scanning calorimetry (DSC) tests, was chosen:

(3) $$\frac{d\alpha }{dt}=\left(K_{1}\exp\left(-\frac{E_{1}}{RT}\right)+K_{2}\exp\left(-\frac{E_{2}}{RT}\right)\cdot \alpha^{m}\right)\cdot {\left(1-\alpha \right)}^{n},$$

where R=8.314 J/mol·K is the universal gas constant, $K_{1}$ and $K_{2}$ are the frequency factors, $E_{1}$ and $E_{2}$ are the activation energies, and n and m are the orders of the reaction. It is necessary to note that the Kamal–Sourour reaction model combines autocatalytic behavior with an *n*-th-order reaction model and besides the first rate constant $K_{1}\exp\left(-E_{1}/RT\right)$ and the exponent n describe the *n*-th-order reaction and the second rate constant $K_{2}\exp\left(-E_{2}/RT\right)$ and the exponent m express the autocatalytic contribution of the reaction. To define the curing kinetic parameters given in Table A3, results of DSC scans performed by Mettler Toledo on samples heated from room temperature to 250 °C at rates of 2, 5, and 10 °C/min and the least squares method fitting the experimental heat flow curves were used.

Optimal results are presented in Table 1 for minimal and maximal ambient temperatures in the industrial shop. It is obvious that all processes were considerably effective for the highest ambient room temperature. For this reason, it is impossible to ignore the effect of ambient temperature on optimization problems of pultrusion processes. The availability of a real technological map for the process producing two rod profiles with ears and operating with a pull speed of 20 cm/min and a control temperature of 135 °C on electrical heaters made it possible to estimate the process effectiveness and productivity. Its energy consumption is given also in Table 1 in square brackets for different ambient shop temperatures. It was seen now that with an application of the developed optimization methodology, the pull speed of this technological process increased by 2.0–2.3 times and the energy consumption reduced by 1.37–1.49 times per 1 m of pultruded profile in dependence on the ambient temperature in the industrial shop.

## 3. Development of Microwave-Assisted Pultrusion Processes

Innovative microwave-assisted pultrusion processes can be described by the following coupled electromagnetic-thermo-chemical problem:

(4) $$\left\{\begin{array}{c} \begin{array}{l} \rho \;c\;\frac{\partial T}{\partial t}-\frac{\partial }{\partial x}\left(k_{x}\frac{\partial T}{\partial x}\right)-\frac{\partial }{\partial y}\left(k_{y}\frac{\partial T}{\partial y}\right)-\frac{\partial }{\partial z}\left(k_{z}\frac{\partial T}{\partial z}\right)-Q_{cd}=0 \end{array}\\ \bar{\rho }\;\bar{c}\;\left(\frac{\partial T}{\partial t}+u\frac{\partial T}{\partial x}\right)-\frac{\partial }{\partial x}\left({\bar{k}}_{x}\frac{\partial T}{\partial x}\right)-\frac{\partial }{\partial y}\left({\bar{k}}_{y}\frac{\partial T}{\partial y}\right)-\frac{\partial }{\partial z}\left({\bar{k}}_{z}\frac{\partial T}{\partial z}\right)-q-Q_{comp}=0\\ \begin{array}{l} \left(\frac{\partial \alpha }{\partial t}+u\frac{\partial \alpha }{\partial x}\right)=R_{r} \end{array} \end{array}\right.,$$

where ρ and c are the density and specific heat of the tooling materials, respectively; $k_{x}$, $k_{y}$, and $k_{z}\;$ are the thermal conductivities of the tooling materials in x, y, and z directions, respectively; u is the pull speed; $\bar{\rho }\;$ and $\bar{c}$ are the lumped density and specific heat for the composite material, respectively; ${\bar{k}}_{x}$, ${\bar{k}}_{y}$, and ${\bar{k}}_{z}\;$ are the lumped thermal conductivities of the composite material in x, y, and z directions, respectively; $R_{r}$ is the rate of the resin reaction; q is the generative term related to the internal heat generation due to the exothermic resin reaction; $Q_{comp}$ is the absorbed energy field in the composite material; and $Q_{cd}$ is the absorbed energy field in the ceramic die, taken into account only for high dielectric losses of the ceramic material. It is necessary to note that the first equation in Equation (4) presents the energy equation for the tool; second, the energy equation for the composite moving in the pull direction; and third, the species or transport equation for the resin. The energy and transport equations are coupled since an exothermic heat release term appears in the energy equation of the moving composite.

**Figure 3:**
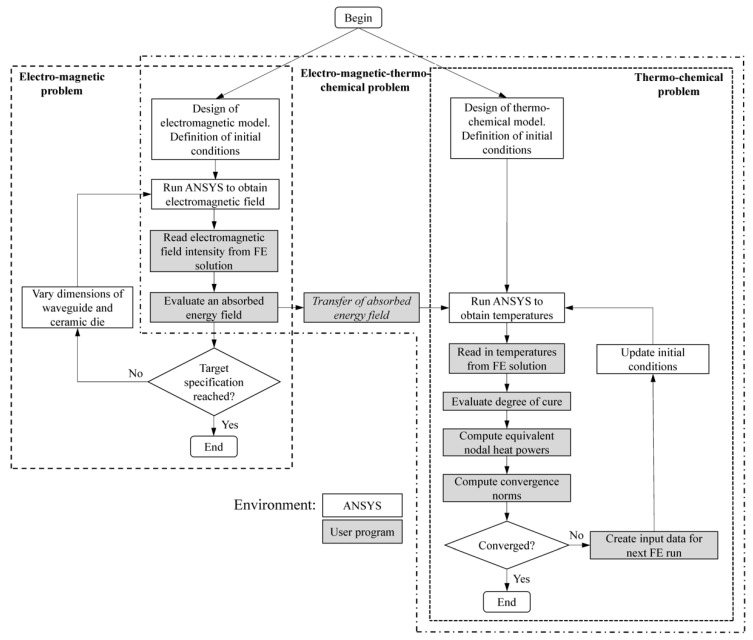
Electromagnetic-thermo-chemical algorithm for a simulation of microwave-assisted pultrusion processes.

The absorption energy fields were determined solving the well-known Maxwell’s equations [12] by the following expression:

(5) $$Q=2\pi f\varepsilon_{0}\varepsilon^{\prime \prime }E_{rms}^{2},$$

where f is the microwave frequency, $\varepsilon_{0}$ is the vacuum permittivity, $\varepsilon^{\prime \prime }$ is the loss factor of electric permittivity, and $E_{rms}$ is the root mean square of the electric field. The system of Equation (4) was solved in ANSYS by the developed algorithm presented in Figure 3. The effectiveness of the microwave heating in pultrusion processes was estimated later as follows:

(6) $$Q_{\%}=\frac{\sum_{i=1}^{N}Q_{i}V_{i}}{P_{MW}}\cdot 100\%,$$

where $Q_{i}$ is the energy absorbed in the *i*-th finite element, $V_{i}$ is the volume of the *i*-th finite element, $P_{MW}$ is the energy of the microwave heating source, and *N* is the number of finite elements used for the modeling of the ceramic die or composite.

**Figure 4:**
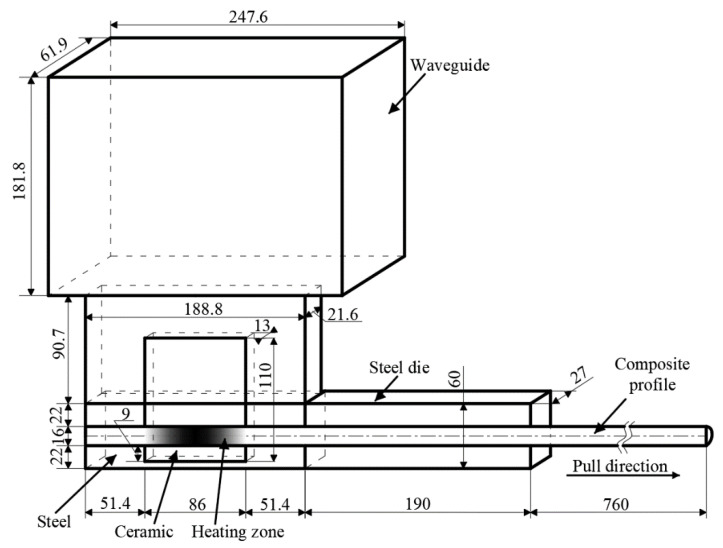
Scheme of microwave-assisted pultrusion process (symmetry used).

**Figure 5:**
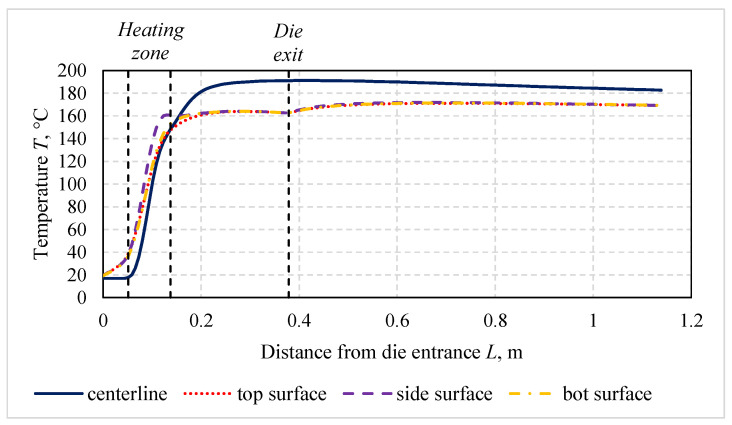
Distribution of temperature along the profile.

**Figure 6:**
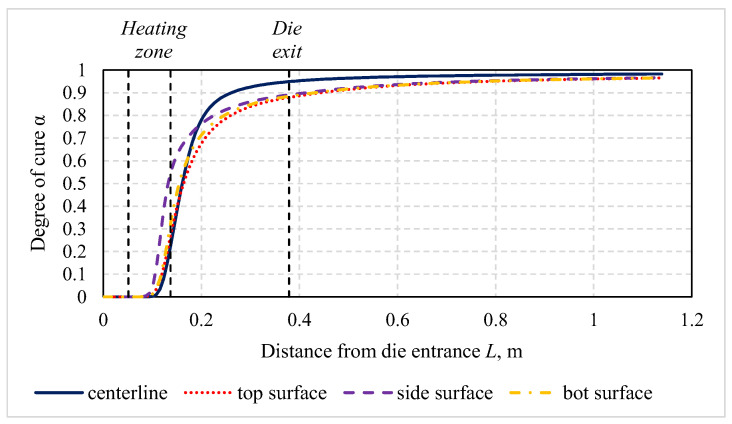
Distribution of the degree of cure along the profile.

To demonstrate the effectiveness and productivity of microwave-assisted pultrusion processes in comparison with conventional processes, the technological process producing a rod profile with a diameter of 16 mm and made of polyester resin POLRES 305BV [36] and glass fibers 4800 tex with a resin mass fraction of 26% was examined. The corresponding thermal material properties are given in Table A1 and Table A2, the kinetic parameters of the resin in Table A3, and the dielectric properties of materials in Table A4, where $\mu_{r}$ is the relative permeability, ${\varepsilon^{\prime }}_{r}$ is the relative permittivity, $\varepsilon_{r}^{\prime \prime }$ is its loss factor, and $R_{0}$ is the resistivity. The innovative pultrusion setup for microwave heating (Figure 4) consisted of a steel die and a microwave block attached to the die. This block included the ceramic inlet made of boron nitride and a waveguide. For the generation of microwaves, a magnetron with a frequency of 2.45 GHz was used. The distribution of temperature and the degree of cure along the profile for the ambient room temperature of 17 °C, a pull speed of 100 cm/min, an applied energy of 0.85 kW, and time of 30 min from the process start are presented in Figure 5 and Figure 6, respectively. The energy consumption of the process in this case was equal to 14.2 W/m.

The numerical study demonstrated that the pull speed of the developed innovative process was higher by 5.6 times and the energy consumption was lower by 1.7 times in comparison with the real conventional process performed at a pull speed of 18 cm/min with an energy consumption of 24.8 W/m and described in detail in [26]. It is necessary to note that the high effectiveness and productivity of microwave-assisted pultrusion processes were obtained by preserving the high quality of pultruded profiles and without optimization. The application of optimization could improve these values additionally.

## 4. Conclusions

The application of the developed non-direct optimization methodology based on the planning of experiments and the response surface technique and holistic simulation tools developed for the design of innovative microwave-assisted pultrusion processes allowed increasing considerably the effectiveness and productivity of existing conventional pultrusion processes operating in industrial and laboratory shops.

## Figures and Tables

**Table 1:** Results of optimization for different temperatures in the industrial shop.

Profile		2 Rods with Ears		Thin-Walled Rectangular		Corner	
Resin		C-L ISO 112G		Crystic VE 676-03		Resoltech 1401+1407+AC140	
Resin mass fraction		22%		47%		44%	
*W_heater_* (W)		2750		750		2750	
*n_heater_*		2		8		2	
Design variables	*T_room_* (°C)	10	40	10	40	10	40
	*V_pull_* (cm/min)	40.9	45.0	48.6	48.5	22.1	26.6
	*T_cont_* (°C)	149	142	-	-	140	136
	*T_cont 1_* (°C)	-	-	140.0	140.0	-	-
	*T_cont 2_* (°C)	-	-	131.6	115.7	-	-
	*X_heater_* (m)	-	-	-	-	0.76	0.76
Objective function	*E* (W/m)	24.3 [33.4]	16.2 [24.1]	77.1	52.1	34.2	22.3

## Data Availability

The data presented in this study are available on request from P.A.

## Appendix A

**Table A1:** Thermal properties of tool materials.

Property	Steel (Conventional Die)	Aluminum (Heater)	Steel (Advanced Die)	Boron Nitride (Ceramic Die)
*ρ* (kg/m^3^)	7720	2700	7850	2100
*c* (J/(kg·K))	470	896	460	960
*k* (W/(m·K))	43	180	33	35

**Table A2:** Thermal properties of composite materials.

Property	Tex 4800	C-L ISO 112G		Crystic VE 676-03		Resoltech 1401+1407+AC140		Polres 305BV	
		Phys. (Resin)	Lumped (Composite)	Phys. (Resin)	Lumped (Composite)	Phys. (Resin)	Lumped (Composite)	Phys. (Resin)	Lumped (Composite)
*ρ* (kg/m^3^)	2500	1100	1953	1100	1564	1200	1992	1100	1870
*c* (J/(kg·K))	1235	1360	1263	1190	1214	1300	1264	1360	1268
*k_x_* (W/(m·K))	11	0.209	0.890	0.188	0.392	0.214	0.475	0.209	0.750
*k_y_* (W/(m·K))	1	0.209	0.546	0.188	0.330	0.214	0.382	0.209	0.500
*T*_max_ (°C)		160		160		185		200	

**Table A3:** Kinetic parameters of resins.

Property	C-L ISO 112G	Crystic VE 676-03	Resoltech 1401+1407+AC140	Polres 305BV
*H_tr_*, (J/kg)	223,385	270,105	334,093	323,074
*K*_1_ (s^−1^)	2.6 × 10^13^	2.98 × 10^11^	3.03 × 10^11^	14,289,310,986
*K*_2_ (s^−1^)	1.2 × 10^12^	6.10 × 10^11^	12,000	285.870
*E*_1_ (J/mol)	116,769	110,865	104,845	85,573
*E*_2_ (J/mol)	200,000	93,241	2,000,000	33,141
*n*	1.27	1.63	0.79	2.342
*m*	0.0011	1.01	0.001	0.519

**Table A4:** Dielectric properties of materials.

Property	Composite	Air	Boron Nitride	Steel
*µ_r_*	1	1	1	1
*ε’_r_*	5.7	1	3.0	1
*ε″_r_*	0.32	0	0.0001	-
*R*_0_ (Ω)	-	∞	-	0
